# Disparities in Treatment Outcomes for Cannabis Use Disorder Among Adolescents

**DOI:** 10.3390/pediatric17040074

**Published:** 2025-07-10

**Authors:** Helena Miranda, Jhon Ostanin, Simon Shugar, Maria Carmenza Mejia, Lea Sacca, Mitchell L. Doucette, Charles H. Hennekens, Panagiota Kitsantas

**Affiliations:** 1 Charles E. Schmidt College of Medicine, Florida Atlantic University, Boca Raton, FL 33431, USA; hdeazeredomi2023@health.fau.edu (H.M.); sshugar2018@health.fau.edu (S.S.); mejiam@health.fau.edu (M.C.M.); lsacca@health.fau.edu (L.S.); chenneke@health.fau.edu (C.H.H.); 2 Herbert Wertheim College of Medicine, Florida International University, Miami, FL 33199, USA; josta002@fiu.edu; 3 Health Economics and Outcomes Research Department, Leafwell, Miami, FL 33156, USA; mdoucette@leafwell.com; 4 Department of Health Administration and Policy, College of Public Health, George Mason University, Fairfax, VA 22030, USA

**Keywords:** cannabis use disorder, adolescent substance use, mental health, substance use treatment

## Abstract

**Background:** This study examined treatment outcomes for cannabis use disorder (CUD) among adolescents (12–17 years old) in the United States. **Methods:** Data from the 2018–2021 Treatment Episode Data Set-Discharges (TEDS-D) included 40,054 adolescents diagnosed with CUD. Descriptive statistics, Chi-square tests, and multivariable logistic regression assessed treatment outcomes and factors associated with treatment completion. **Results:** Only 36.8% of adolescents completed treatment. The most common reasons for not completing treatment were dropping out (28.4%) and transferring to another facility/program (17.0%). Males and Black non-Hispanic adolescents had lower odds of completing treatment (OR = 0.79, 95%CI: 0.75–0.84), while Hispanic (OR = 1.13, 95%CI: 1.08–1.18), Asian (OR = 1.56, 95%CI: 1.3–1.86) and Native Hawaiian/Pacific Islander adolescents (OR = 2.31, 95%CI: 2.04–2.61) had higher odds of completion compared to their White counterparts. Independent living arrangements, homelessness, arrests in the past 30 days and younger age (<15 years old) decreased the likelihood of treatment completion. Adolescents with co-occurring mental health and substance use disorders also had lower completion rates (OR = 0.79, 95%CI: 0.77–0.86). Referral from schools/employers and treatment settings were associated with a higher success, particularly with stays of 4–6 months and 7–12 months. **Conclusion:** This study highlights the need for targeted CUD treatment programs that support at-risk adolescents, especially those experiencing homelessness or facing legal issues. High dropout and transition rates suggest a need for continuity of care and program integration between facilities. Strengthening coordination among public health officials, community organizations, and stakeholders is essential to developing culturally responsive treatment interventions that address social determinants of health, substance use, and mental health in this vulnerable population.

## 1. Introduction

In recent years, cannabis or marijuana use among United States (US) adolescents has been decreasing. Current evidence suggests that the percentage of adolescents reporting current marijuana use fell significantly from 23.1% in 2011 to 15.8% in 2021 [1]. Additionally, the percentage of those who tried marijuana for the first time before age 13 decreased from 8.1% in 2011 to 4.9% in 2021. Current use showed similar significant overall declines as well as in various racial and ethnic groups. For example, Asian, Hispanic, and White adolescents experienced the most notable reductions [1]. Among US young adults (19 to 30 years old), however, daily use peaked in 2022 at 11%, surpassing levels from both five years ago (8% in 2017) and ten years ago (6% in 2012) [2]. Daily or regular use can increase the risk of cannabis use disorder (CUD). CUD, defined as a pattern of cannabis use leading to significant impairment or distress, includes persistent cravings, inability to control use, and withdrawal symptoms [3], has been reported to be more prevalent in adolescents than adult cannabis users [4].

Adolescence is a critical period for brain development, and cannabis use during this time can have detrimental long-term effects on cognitive function, academic performance, and social outcomes. Regular cannabis use in adolescents is linked with impaired memory, attention, and executive function which can hinder academic achievement and increase the risk of school dropout [5]. Furthermore, early cannabis use has been associated with poorer social outcomes, including reduced life satisfaction and lower socioeconomic status in adulthood [6]. Frequent teenage cannabis use shows a more consistent correlation with adverse functional outcomes at age 20 than frequent teenage nicotine or alcohol use [7]. These findings highlight the importance of addressing adolescent cannabis use and CUD proactively to mitigate long-term consequences.

Currently, reliable evidence is sparse regarding the effectiveness in adolescent populations of treatment approaches to CUD. Evidence from randomized controlled trials and real-world observational studies has highlighted the efficacy of behavioral interventions, such as cognitive behavioral therapy, motivational enhancement therapy, and contingency management, in reducing cannabis use and improving abstinence rates among adolescents and adults [8,9]. However, challenges remain in scaling these interventions to real-world settings. In addition to these challenges, observational studies indicate that as cannabis use has increased, treatment admissions have declined [10]. This inverse relationship, even when accounting for variables such as health insurance coverage and treatment center availability, is compatible with the hypotheses that increased social acceptance and decreased perception of harm associated with cannabis use may deter individuals from seeking treatment [10]. If so, this shift could lead to a growing unmet need for CUD treatment with adolescents potentially delaying or avoiding intervention until their substance use becomes more severe. Developing strategies to maintain awareness of the risks associated with cannabis use, despite its increasing social acceptance, seems crucial for encouraging early intervention.

In addition, understanding the nuances of CUD-related treatments and their outcomes is essential in informing the development of more effective, targeted interventions. Given the growing concerns about the adverse effects of cannabis use on adolescent health and the lack of current information on CUD treatments in this population, the present study sought to examine CUD treatment outcomes, with a focus on identifying factors associated with treatment completion and disparities in this population. By identifying key predictors and disparities, the findings can guide the development of targeted interventions to improve treatment outcomes for adolescents struggling with CUD.

## 2. Materials and Methods

### 2.1. Data and Study Sample

We utilized data from the 2018–2021 Treatment Episode Data Set-Discharges (TEDS-D). TEDS-D is a US national data system that includes records on admissions of people aged 12 and older to substance use treatments. Data were collected on all discharges from state-licensed or certified substance use treatment facilities that receive federal funding and report their data to the Substance Abuse and Mental Health Administration [11]. All states provide information that includes demographics, substance use, living arrangements, prior arrests, referral source, number of prior treatment episodes, type of treatment received and other characteristics at the time of discharge. Federally funded facilities operate under federal guidelines to ensure accessibility for vulnerable populations, often regardless of insurance status. Adolescents access these services through referrals from schools, courts, health care providers, or self-referral. These programs frequently integrate behavioral interventions tailored to youth development needs. More information about the TEDS-D can be found elsewhere [11].

The analytic sample was restricted to discharges of patients between the ages of 12 and 17 years who were diagnosed with cannabis abuse and/or cannabis dependence based on DSM-IV criteria [3]. The sample was further limited to cases without a history of prior substance use treatment, ensuring that each case was considered as an individual rather than a discharge occurrence from a treatment facility. The TEDS data do not contain unique identifiers that can track patients longitudinally. The final sample comprised 40,054 adolescents diagnosed with CUD and with no prior treatment episodes. The missing data for variables ranged from <0.001% to 8.4%. Since the missing data analysis indicated that the data were missing completely at random, a complete case analysis was conducted. This approach did not significantly reduce the sample size or compromise statistical power. Since the study utilized publicly available data, it qualified for exemption from review by the Institutional Review Board.

### 2.2. Measures

TEDS-D data contain information on treatment outcomes which were captured in the variable ‘reason for discharge,’ and included (1) treatment completed, (2) dropped out of treatment, (3) terminated by facility, (4) transferred to another treatment program or facility, (5) incarcerated, (6) death, and (7) other [11]. In this study, we recoded this variable into either “treatment completed” or “treatment not completed” which included those who dropped out of treatment, were terminated by the facility, transferred to another treatment program or facility, died, were incarcerated, and other reasons. Treatment completion is defined as instances where all parts of the prescribed treatment plan or program were fully completed, as documented at discharge in the TEDS-D data set.

The independent measures included demographic variables, arrests, living arrangements, and treatment-related measures. Demographic measures were gender (male and female), age at admission (12–14 years and 15–17 years), race/ethnicity (White non-Hispanic, Black non-Hispanic, Hispanic, American Indian/Alaskan Native, Native Hawaiian/Asian Pacific Islander, Asian, and Other). Living arrangements identified the housing status of adolescents at the time of discharge (homeless, dependent living, and independent living), and the number of arrests within 30 days prior to discharge (none, one, and ≥2). Age of first use of cannabis (11 years and under, 12–14 years, and 15–17 years), co-occurring mental and substance use disorders at discharge (yes or no) and co-substance use at admission (yes or no) were included as well.

Treatment-related measures included, referral source (individual/self-referral, alcohol/drug use care provider, other health care provider, school, employer/employee assistance program (EAP), other community referral, and court/criminal justice), treatment service/setting at discharge (detox 24 h, rehab/residential short-term, rehab/residential long-term, ambulatory intensive outpatient, and ambulatory non-intensive outpatient), length of stay (1 month or less, 2–3 months, 4–6 months, 7–12 months, and more than 12 months), and year of discharge from treatment (2018, 2019, 2020, 2021). Drug service settings in TEDS-D refer to the type of treatment program provided. For example, residential rehabilitation involves round-the-clock care, whereas outpatient services allow patients to remain at home while attending regular treatment sessions.

### 2.3. Statistical Analysis

Descriptive statistics were conducted to examine trends and sample characteristics. Chi-square tests were used to determine whether there were significant differences in treatment completion across the sample characteristics (e.g., demographics, living arrangements, arrests, treatment service/setting, length of stay, etc.). Multivariable logistic regression analysis was performed to assess factors that influence CUD treatment completion in adolescents. Results were expressed as odds ratios (ORs) with 95% Confidence Intervals (CI). All *p*-values were two sided and we considered *p*-values < 0.05 as significant. The statistical analyses were conducted using SPSS software (version 29) [12].

## 3. Results

### 3.1. Sample Characteristics

In this sample, most of the adolescents were male (71.4%) and 81.4% were 15–17 years old (Table 1). Approximately 43.0% were White non-Hispanic followed by 23.0% Black non-Hispanic and Hispanic. Over 60% reported dependent living, 9.2% had at least one arrest in the past 30 days, 59.0% started using cannabis at age 12–14 years old, and over one-third reported co-occurring mental health and substance use disorders. The primary referral source was the judicial system (38.5%), individual/self-referral (20.3%), and other health care providers (14.8%), while ambulatory, non-intensive outpatient was the most common treatment setting (72.1%). Most adolescents spent 2–6 months in treatment.

### 3.2. Treatment Outcomes for CUD

**Figure 1:**
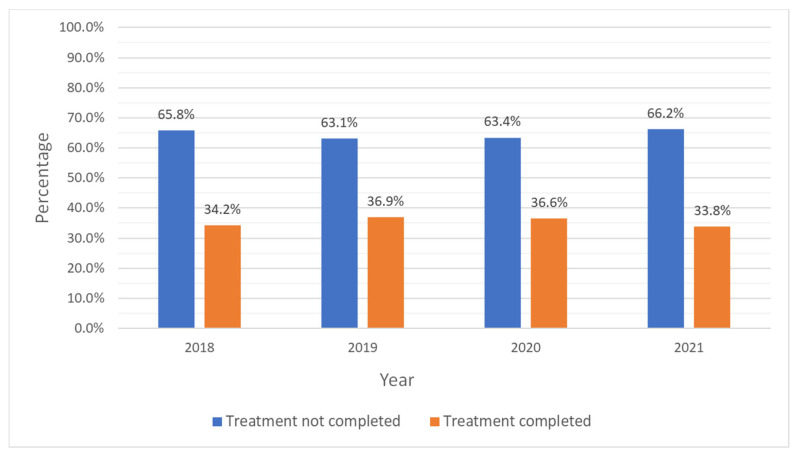
Trends in treatment completion status for cannabis use disorder in adolescents: 2018–2021 Treatment Episode Data Set-Discharges (TEDS-D).

**Figure 2:**
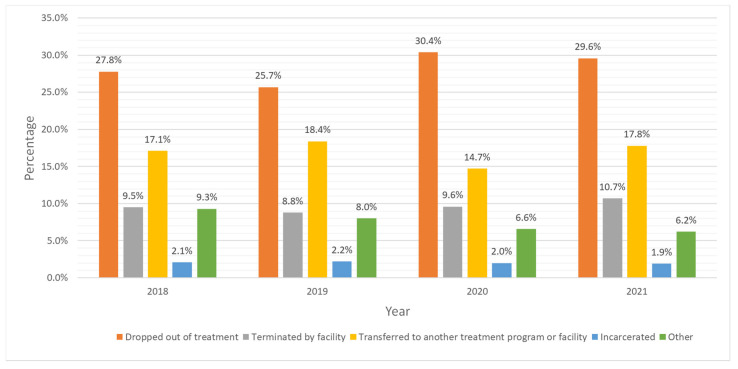
Trends in treatment outcomes for cannabis use disorder among adolescents who did not complete treatment, 2018–2021 Treatment Episode Data Set- Discharges (TEDS-D).

In this sample, only 36.8% of adolescents with CUD completed their treatment (Table 1). Figure 1 shows that treatment completion remained about the same from 2018 to 2021, ranging from 36.9% in 2019 to 33.8% in 2021, which constituted the lowest completion percentage in this period. The most common reasons for not completing treatment in all years were dropping out of treatment (28.4%), transferring to another facility (17.0%), and being terminated by the facility (9.7%) (Figure 2).

### 3.3. CUD Treatment Outcomes and Sample Characteristics in Bivariable Analysis

Gender and age differences in treatment completion were minimal, with males and younger adolescents having a lower likelihood of completing treatment (Table 1). Hispanic adolescents and those identifying as Native Hawaiian/Pacific Islander/Asian Pacific Islander were more likely to complete treatment. In contrast, Black and White non-Hispanic adolescents were less likely to complete treatment. Adolescents in dependent living arrangements had significantly higher completion rates (64.4%), while it was significantly lower for those in independent living (35.3%) or homeless situations (0.2%) (*p* < 0.001). In addition, a history of arrests in the past 30 days was associated with lower treatment completion rates. Adolescents who began using cannabis at 11 years or younger had a significantly lower completion rate (12.9%), while it was higher for those who started at 15–17 years (*p* < 0.001). Adolescents with co-occurring mental and substance use disorders (30.3%) and the use of substances other than cannabis at admission (31.9%) were less likely to complete treatment.

Those referred from employers/EAPs and schools had significantly higher completion rates. Furthermore, adolescents whose treatment service/setting was either short or long-term rehab/residential had a higher likelihood of completing treatment (4.4% and 9.5%, respectively). Adolescents who stayed in treatment for 4–6 months had higher completion rates (37.8%), while those with stays longer than 12 months showed no marked difference. Adolescents staying for less than 1 month had the lowest completion rates (9.3% for completed treatment versus 33.6% for not completed).

### 3.4. CUD Treatment Completion and Sample Characteristics in Multivariable Analyses

Table 2 shows the ORs and 95% CI for associations between CUD treatment completion and sample characteristics. Males had a slightly lower likelihood of completing treatment compared to females (OR = 0.95, 95% CI: 0.91–0.99). Regarding racial and ethnic differences, Black non-Hispanic adolescents had significantly lower odds of treatment completion (OR = 0.79, 95% CI: 0.75–0.84) relative to their White counterparts. In contrast, Native Hawaiian/Pacific Islander/Asian Pacific Islander (OR = 2.31, 95% CI: 2.04–2.61), Hispanic (OR = 1.13, 95% CI: 1.08–1.19), and Asian (OR = 1.56, 95% CI: 1.31–1.86) adolescents had higher odds of completing treatment compared to White non-Hispanic adolescents. Adolescents in independent living (OR = 0.82, 95% CI: 0.79–0.86) or homeless (OR = 0.50, 95% CI: 0.35–0.73) situations had lower odds of treatment completion compared to those in dependent living arrangements. Additionally, having one and ≥2 arrests in the past 30 days was associated with significantly lower odds of treatment completion (OR = 0.33, 95% CI: 0.29–0.36; OR = 0.24, 95%, CI: 0.19–0.29, respectively) compared to adolescents with no arrests.

Adolescents who began using cannabis at 11 years or younger had a significantly lower likelihood of treatment completion (OR = 0.66, 95% CI: 0.62–0.71) than those between 15 and 17 years of age. Adolescents with co-occurring mental and substance use disorders had lower odds of treatment completion (OR = 0.79, 95% CI: 0.77–0.84) relative to those without co-occurring mental and substance use disorders. Co-substance use at admission was also associated with significantly lower odds of treatment completion (OR = 0.84, 95% CI: 0.81–0.88).

Referrals from employers/EAP (OR = 3.75, 95% CI: 3.06–4.59) and school (OR = 1.16, 95% CI: 1.08–1.24) were associated with significantly higher odds of treatment completion, while self-referrals (OR = 0.82, 95% CI: 0.77–0.86) and referrals from alcohol/drug use care providers (OR: 0.76, 95% CI: 0.68–0.84) were associated with lower odds of treatment completion compared to court/criminal justice referrals. Adolescents in detox (OR = 2.89, 95% CI: 2.44–3.44), short-term (OR = 2.06, 95% CI: 1.88–2.25), or long-term (OR = 2.69, 95% CI: 2.50–2.90) residential settings were significantly more likely to complete treatment than those in an ambulatory, non-intensive outpatient setting. A stay of 4–6 months and 7–12 months was associated with higher odds of treatment completion (OR = 1.19, 95% CI: 1.08–1.32; OR = 1.26, 95% CI: 1.13–1.39, respectively), while stays of 1 month or less were associated with significantly lower odds (OR = 0.18, 95% CI: 0.17–0.21) when compared to >12 months. Adolescents discharged in 2020 (OR = 0.83, 95% CI: 0.79–0.88) and 2021 (OR = 0.90, 95% CI: 0.85–0.96) had significantly lower odds of completing treatment compared to those in 2018.

## 4. Discussion

From 2018 to 2021, completion rates in US adolescents for CUD aged 12 to 17 years of age were similar and low. Specifically, the percentages for completion rates ranged from 36.9% in 2019 to 33.8% in 2021. The most common reasons for not completing treatment in all years were dropping out of treatment, transferring to another facility, and being terminated by the facility. These findings reflect well-documented challenges in adolescent substance use treatment [13] and are compatible with prior research indicating that traditional treatment approaches have not yielded satisfactory retention and abstinence rates. For instance, in a study evaluating adaptive treatment (AT) strategies for adolescents who struggled to achieve abstinence, only 37% of them completed the AT phase, and just 27% successfully achieved abstinence [14]. Both studies highlight the need for innovative, integrative approaches to enhance treatment efficacy. Incorporating wraparound services, addressing social determinants of health, and integrating family and community-based interventions may improve engagement and outcomes in this population.

In addition, the findings of the present study show that males were less likely to complete treatment than females. However, the narrowing gender gap in cannabis use as it becomes more socially accepted among females may complicate these trends [1,15]. Black and White adolescents also had lower completion rates. These patterns align with national data indicating higher cannabis use among males and Black adolescents but lower access to and completion of treatment [16,17]. Despite the overall increase in recreational cannabis use following legalization [18], Black and White adolescents may face unique barriers that limit their likelihood of success in treatment programs in conjunction with likely a wider acceptance of cannabis as a social drug. These disparities highlight the urgent need for tailored interventions that address the unique socioeconomic and cultural needs and beliefs and acceptability of cannabis in these racial/ethnic populations as perhaps a safe drug.

Furthermore, adolescents in dependent living arrangements had higher treatment completion rates, while those in independent or homeless situations showed significantly lower success rates. Youth experiencing homelessness face disproportionately high rates of mental health and substance use challenges, making treatment engagement and completion more difficult [13,19,20]. These findings further emphasize the need for tailored interventions that address both housing stability and early prevention strategies, particularly for vulnerable youth experiencing homelessness and comorbid mental health issues. Ensuring access to stable housing and providing early interventions could improve treatment engagement and outcomes for these at-risk populations [21].

Early cannabis use also emerged as a strong predictor of lower treatment completion. Adolescents who begin using cannabis at younger ages tend to develop more severe substance use disorders, which compounds the challenges in completing treatment. Research consistently links early cannabis initiation with worse cognitive, psychological, and social outcomes [4], which further complicates recovery efforts. These findings highlight the critical need for earlier intervention to prevent substance use initiation, particularly for cannabis. Evidence shows that a significant proportion of adults admitted to substance use treatment began using substances as early as age 11 or younger [22]. Adolescents who started using cannabis before age 15 had notably lower treatment completion rates, underscoring the importance of beginning prevention efforts in elementary or middle school.

Existing prevention programs that focus on “zero tolerance” have proven ineffective, highlighting the need for culturally relevant, evidence-based alternatives that are both inclusive and multifaceted [23,24]. Approaches that incorporate parent training and community involvement have shown promising results in early intervention efforts. Investing in research to develop and test such interventions is critical, especially for younger children, and should be sustained through middle and high school, when substance use typically accelerates [25]. Engaging parents, families, and communities in prevention initiatives, as well as communicating the importance of early intervention to policymakers and stakeholders, is key to expanding and improving these efforts. Comprehensive strategies addressing these needs could help reduce the number of adolescents who develop severe substance use disorders, improving treatment outcomes in later years.

Referral sources played a significant role in treatment outcomes in this study, with adolescents referred by schools, employers, or the legal system exhibiting higher completion rates compared to those who self-referred. This may reflect external pressures, such as compliance with school or court requirements, which can motivate adherence. However, such pressures do not necessarily foster intrinsic motivation or ensure long-term recovery. Leveraging these external support systems while simultaneously addressing internal motivators for sustained behavioral change is essential for improving outcomes. This underscores the critical importance of external support structures, particularly in environments such as schools, where adolescents can be monitored more closely. The findings highlight the value of strengthening collaborations between educational institutions and treatment providers, which could improve both treatment retention and overall success for adolescents with CUD. By funding school-based treatment programs and fostering partnerships between education and health care systems, these collaborations can ensure more effective referral processes and tailored interventions that better meet adolescents’ needs.

Furthermore, one of the key factors influencing treatment outcomes was the length of stay. In particular, stays ranging from 4 to 12 months were linked to a higher likelihood of completing CUD treatment. Similar findings have been observed in previous studies in non-adolescent populations [26] while others indicate that 90 days is the minimum recommended duration for treatment across substances [13]. As cannabis becomes more widely available, the demand for longer, more effective, and potentially more comprehensive treatments for CUD, especially for vulnerable populations such as adolescents, is likely to grow [26].

This study has some unique advantages. The data derive from a large, nationally representative sample of clients. This enhances the generalizability of our findings across diverse adolescent populations. However, several limitations must be noted. The reliance on self-reported data may introduce biases such as underreporting and social desirability bias. In addition, the data set only includes facilities receiving public funding, limiting the generalizability to private treatment centers or other settings. The data set focuses on discharge outcomes, lacking insight into long-term recovery and relapse rates, which are critical to fully understanding treatment success. The absence of detailed information on certain outcomes, such as the specific reasons (e.g., personal, systemic, or logistical barriers) for dropping out of treatment, restricts our ability to gain deeper insights into treatment completion. The data set also does not have details on therapeutic interventions and medications used, limiting the ability to evaluate treatment approaches, such as behavioral therapies or pharmacological treatments, relative to treatment completion. While cannabis use frequency at admission and discharge is captured, significant missing data, particularly in adolescents, prevented its inclusion in this analysis. Moreover, treatment completion is widely considered a critical milestone, as it is associated with better post-treatment outcomes in other substance use disorders. However, the lack of follow-up or longitudinal data on post-discharge relapse or functional outcomes limits the scope of conclusions we can draw. Finally, this study lacks data on key psychosocial vulnerabilities such as trauma history, adverse childhood experiences, family dynamics, peer influence, educational challenges, and access to social support systems, all of which are critical in shaping the trajectory of treatment outcomes in young people.

These limitations in the present study underscore the need for future research that includes longitudinal data to evaluate sustained recovery and relapse rates among adolescents, as well as more comprehensive data on treatment modalities and cannabis use patterns. In addition, it is important to gather detailed reliable data on cannabis use, including frequency, type of product, quantity, and methods of consumption. To better understand treatment engagement and outcomes, psychosocial data such as adverse childhood experiences, family support structures, and other social determinants of health should also be collected. These factors are often closely linked to substance use behaviors and treatment success. Investigating the impact of co-occurring mental health disorders, which significantly affect treatment outcomes, is also critical. Lastly, culturally tailored interventions should be prioritized to address treatment disparities and improve retention rates in this vulnerable population.

## 5. Conclusions

This study provides valuable insights into the factors influencing treatment completion for CUD among adolescents, revealing significant disparities across demographic and treatment-related characteristics. The findings underscore the critical role that external support systems, such as schools and legal systems, play in facilitating treatment success. Overall, the impact of early cannabis use and co-occurring mental health disorders further emphasize the complexity of achieving successful outcomes for adolescents with CUD. Furthermore, the consistently lower treatment completion rates among non-Hispanic Black and White adolescents as well as youth experiencing homelessness underscore the need for further research to understand why treatment interventions are less effective for these populations. Future research should also examine treatment programs for adolescent CUD in other industrialized countries as insights gained may help enhance treatment approaches and outcomes in the US. Addressing these multifaceted challenges requires dedicated funding for research initiatives focused on treatment approaches for CUD among vulnerable populations. It also requires comprehensive and culturally relevant strategies to improve treatment access and outcomes for all adolescents.

## Figures and Tables

**Table 1:** Bivariable associations between CUD treatment outcomes and sample characteristics in adolescents with no prior treatment episodes: 2018–2021 Treatment Episode Data Set-Discharges.

Characteristics	Full Sample *n* (%)	Did Not Complete Treatment *n* = 25,324 (63.2%) *n* (%)	Completed Treatment *n* = 14,730 (36.8%) *n* (%)	*p*-Value
**Gender**				0.082
Male	28,586 (71.4)	71.7	70.9	
Female	11,460 (28.6)	28.3	29.1	
**Age at admission**				0.523
12–14 years	7467 (18.6)	18.7	18.5	
15–17 years	32,587 (81.4)	81.3	81.5	
**Race/ethnicity**				<0.001
White, non-Hispanic	16,972 (43.4)	44.8	41.1	
Black, non-Hispanic	8943 (22.9)	24.9	19.4	
Hispanic	8955 (22.9)	21.2	25.8	
American Indian/Alaska Native	723 (1.8)	1.7	2.2	
Native Hawaiian/Pacific Islander/Asian Pacific Islander	1248 (3.2)	1.8	5.6	
Asian	497 (1.3)	1.0	1.8	
Other	1744 (4.5)	4.7	4.1	
**Living arrangements**				<0.001
Homeless	132 (0.4)	0.4	0.2	
Dependent living	22,646 (61.1)	59.0	64.4	
Independent living	14,290 (38.6)	40.6	35.3	
**Arrests in past 30 days**				<0.001
None	34,304 (90.9)	87.5	96.2	
Once	2749 (7.3)	9.9	3.1	
Two or more times	705 (1.9)	2.6	0.7	
**Age at first use of cannabis (years)**				<0.001
11 years and under	5691 (14.4)	15.3	12.9	
12–14	23,258 (58.8)	59.4	57.8	
15–17	10,615 (16.8)	25.4	29.4	
**Co-occurring mental health and substance use disorders**				<0.001
Yes	13,728 (37.4)	41.3	30.3	
No	22,959 (62.6)	58.7	69.7	
**Co-substance use at admission**				<0.001
Yes	13,633 (34.0)	35.3	31.9	
No	26,421 (66.0)	64.7	68.1	
**Referral source**				<0.001
Individual/self-referral	7974 (20.3)	20.9	19.2	
Alcohol/drug use care provider	1356 (3.5)	4.3	2.0	
Other health care provider	5802 (14.8)	14.6	15.1	
School	3866 (9.8)	9.3	10.7	
Employer/EAP	596 (1.5)	0.6	3.2	
Other community referral	4553 (11.6)	12.1	10.7	
Court/criminal justice	15,128 (38.5)	38.2	39.0	
**Treatment service/setting**				<0.001
Detox, 24 h, free-standing residential	333 (0.8)	0.8	0.9	
Rehab/residential, short term (30 days or fewer)	500 (3.7)	3.4	4.4	
Rehab/residential, long term (>30 days)	3236 (8.1)	7.3	9.5	
Ambulatory, intensive outpatient	6084 (15.2)	15.4	14.8	
Ambulatory, non-intensive outpatient	28,883 (72.1)	73.2	70.4	
**Length of stay**				<0.001
1 month or less	9876 (24.7)	33.6	9.3	
2–3 months	11,692 (29.2)	28.5	30.3	
4–6 months	11,298 (28.2)	22.7	37.8	
7–12 months	5513 (13.8)	11.3	18.1	
>12 months	1675 (4.2)	4.0	4.5	

**Table 2:** Logistic regression assessing associations between cannabis use disorder treatment completion and sample characteristics in adolescents with no prior treatment episodes: 2018–2021 Treatment Episode Data Set-Discharges.

Characteristics	Treatment Completion Odds Ratio (95% Confidence Interval)
**Gender**	
Male	**0.95 (0.91–0.99)**
Female	Reference
**Age at admission**	
12–14 years	0.98 (0.93–1.04)
15–17 years	Reference
**Race/ethnicity**	
White, non-Hispanic	Reference
Black, non-Hispanic	**0.79 (0.75–0.84)**
Hispanic	**1.13 (1.08–1.18)**
American Indian/Alaska Native	1.12 (0.98–1.28)
Native Hawaiian/Pacific Islander/Asian Pacific Islander	**2.31 (2.04–2.61)**
Asian	**1.56 (1.31–1.86)**
Other	**0.88 (0.79–0.96)**
**Living arrangements**	
Homeless	**0.50 (0.35–0.73)**
Dependent living	Reference
Independent living	**0.82 (0.79–0.86)**
**Arrests in the past 30 days**	
None	Reference
Once	**0.33 (0.29–0.36)**
Two or more times	**0.24 (0.19–0.29)**
**Age at first use of cannabis (years)**	
11 years and under	**0.66 (0.62–0.71)**
12–14	**0.79 (0.75–0.83)**
15–17	Reference
**Co-occurring mental and substance use disorders**	
Yes	**0.79 (0.77–0.84)**
No	Reference
**Co-substance use at admission**	
Yes	**0.84 (0.81–0.88)**
No	Reference
**Referral source**	
Individual/self-referral	**0.82 (0.77–0.86)**
Alcohol/drug use care provider	**0.76 (0.68–0.84)**
Other health care provider	**0.89 (0.84–0.95)**
School	**1.16 (1.08–1.24)**
Employer/EAP	**3.75 (3.06–4.59)**
Other community referral	**0.86 (0.81–0.92)**
Court/criminal justice	Reference
**Treatment service/setting**	
Detox, 24 h, free-standing residential	**2.89 (2.44–3.44)**
Rehab/residential, short term (30 days or fewer)	**2.06 (1.88–2.25)**
Rehab/residential, long term (>30 days)	**2.69 (2.50–2.90)**
Ambulatory, intensive outpatient	1.01 (0.95–1.07)
Ambulatory, non-intensive outpatient	Reference
**Length of stay**	
1 month or less	**0.18 (0.17–0.21)**
2–3 months	**0.68 (0.61–0.75)**
4–6 months	**1.19 (1.08–1.32)**
7–12 months	**1.26 (1.13–1.39)**
>12 months	Reference
**Year of discharge**	
2018	Reference
2019	0.97 (0.93–1.02)
2020	**0.83 (0.79–0.88)**
2021	**0.90 (0.85–0.96)**

**Bolded** results indicate significant findings at *p*-value < 0.05.

## Data Availability

The data presented in the study are openly available in the Treatments Episode Data Set Discharges at https://www.samhsa.gov/data/data-we-collect/teds-treatment-episode-data-set (accessed on 3 June 2024).
